# Thioredoxin *h1*-targeted proteins in European beech (*Fagus sylvatica* L.): Insights into metabolic control, stress response, and seed longevity

**DOI:** 10.1371/journal.pone.0359622

**Published:** 2026-10-05

**Authors:** Ewelina Ratajczak, Aleksandra M. Staszak, Paola Vargas, Mariam Sahrawy, Antonio J. Serrato, Hanna Fuchs

**Affiliations:** 1 Institute of Dendrology, Polish Academy of Sciences, Parkowa, Kórnik, Poland; 2 Laboratory of Plant Physiology, Department of Plant Biology and Ecology, Faculty of Biology, University of Białystok, Ciołkowskiego, Białystok, Poland; 3 Department of Stress, Development and Signaling in Plants, Estación Experimental del Zaidín, Consejo Superior de Investigaciones Científicas (CSIC), Granada, Spain; The University of Tokyo: Tokyo Daigaku, JAPAN

## Abstract

Seed viability and longevity underpin forest regeneration under changing environmental conditions. European beech (*Fagus sylvatica* L.) produces intermediate seeds that gradually lose viability during storage, largely through oxidative stress, which makes redox homeostasis central to their longevity. Here we investigated the role of thioredoxin *h1* (TRX *h1*), a cytosolic thiol-disulfide oxidoreductase, in the embryonic axes of beech seeds. Using a His-tagged monocysteinic (Cys to Ser) trapping mutant of TRX *h1* combined with data-dependent acquisition (DDA) proteomics, we identified 161 putative TRX *h1* target proteins, which fall into functional categories related to genetic information processing, metabolism, environmental and cellular signaling, and protein homeostasis. Western blotting confirmed that target capture proceeded through DTT-reducible mixed-disulfide complexes, supporting a redox-based interaction. The targets include heat shock proteins (HSP70 and HSP90) and other chaperones, calcium-binding proteins (calmodulin and calreticulin), a methyl-CpG-binding protein, glycolytic and tricarboxylic acid (TCA) cycle enzymes, and antioxidant enzymes such as superoxide dismutase, glutathione peroxidase and glutaredoxin. To link these targets to seed physiology, we quantified the thiol redox status of fresh and long-term stored seeds: total thiols were approximately 2.2-fold lower in stored than in fresh axes (21.0 ± 0.5 vs 45.9 ± 1.3 nmol GSH equivalents per axis), indicating that the reduced thiol pool, the substrate of the TRX *h1* system, becomes depleted and more oxidized during storage. Together, these results suggest that TRX *h1*-dependent redox maintenance may contribute to redox homeostasis, energy metabolism and protein stability in beech embryonic axes, and provide a basis for improving seed storage and conservation strategies.

## 1. Introduction

The long-term stability and regeneration capacity of forests critically depend on the production of viable seeds. Seed viability, germination potential, and storage behavior are influenced by a complex interplay of environmental conditions and intrinsic physiological factors. With an increasing impact of climate change on seed production, germination, and survival, research on forest dynamics has placed seed biology at the center of ecological studies [1]. However, many forest models fail to account for the variability in seed production and often assume continuous seed availability even in the absence of mature trees [2]. Moreover, despite the importance of seed traits in community ecology they remain underexplored [3]. The future structure and resilience of forests are directly linked to the reproductive success of tree species.

Masting, which is a reproductive strategy where tree species produce large seed crops in certain years followed by years of scarcity, is common among long-lived species such as European beech (*Fagus sylvatica* L.). Climate change negatively affects masting behavior, which disrupts seed production across multiple taxa [4,5]. Both excessive and insufficient seed outputs can hamper forest regeneration by limiting reproductive material. Consequently, effective seed storage strategies are increasingly crucial for both commercial purposes and conservation of biodiversity [6,7].

A significant challenge in seed conservation is seed aging during storage. As seeds age, their viability gradually decreases, leading to delayed or failed germination, poor seedling establishment, and even a complete loss of germination potential [8,9]. For species such as European beech, which masts every 5–10 years, proper seed storage is essential for maintaining biodiversity and regeneration capacity. Climate projections indicate that Central Europe will experience more frequent heatwaves and summer droughts, which may affect forest dynamics [10]. At the same time, predictions suggest that the range of European beech could expand in response to climate change, further emphasizing the importance of optimized storage techniques to support forest regeneration and adaptation [11].

European beech seeds are classified as intermediate seeds, which means that they lose viability more quickly than orthodox seeds, but they can tolerate partial dehydration [12,13]. However, these seeds are more susceptible to oxidative stress during storage because their antioxidant systems are less efficient [14]. Previous studies have shown that the deterioration of seed viability is closely associated with the accumulation of reactive oxygen species (ROS) and resulting oxidative stress [15]. This oxidative stress causes modifications to proteins, lipids, and nucleic acids and impairs the cellular machinery necessary for successful germination. Redox-sensitive proteins, such as glyceraldehyde-3-phosphate dehydrogenase (GAPDH), NAD-dependent dehydrogenases, and peptidyl-prolyl cis-trans isomerases, are particularly vulnerable to oxidative modifications during storage. In European beech seeds, protein-bound thiols were previously shown to be highly dynamic during development and storage, with a decline during storage, whereas non-protein thiols remained comparatively stable, indicating that the protein thiol pool is a storage-sensitive component of the seed redox system [16]. In response, proteins, such as thioredoxins (TRXs) and thiol peroxiredoxins (Prxs), play critical roles in maintaining redox balance and protecting cells from oxidative damage. In European beech seeds, the 2-cysteine peroxiredoxin (2-Cys Prx) protein is a marker of seed viability, which reinforces the importance of redox homeostasis for seed longevity [16].

Among the thioredoxin *h* family, TRX *h1* was selected for this study because of its well-characterized role in seed redox regulation in related species. We hypothesize that the target proteins of thioredoxin *h1* (TRX *h1*) are integral to redox homeostasis, energy metabolism, and protein folding and may contribute to seed viability and longevity during storage. TRXs are small redox proteins with an active site comprised of two redox cysteines. When these cysteines are in the form of thiols, they can reduce target proteins and thereby break disulfide bonds; this process usually activates target enzymes [17]. Consequently, the active-site cysteines of TRXs form a disulfide bridge. In turn, TRXs are reduced by NADP-thioredoxin reductase (NTR). Both TRX and NTR proteins constitute the NTR system, which is part of the antioxidant enzymatic system present in the cytosol and nuclei of plant cells [18,19]. The interaction between TRX *h1* and its target proteins modulates cellular processes that protect seeds from oxidative damage and prepare them for metabolic readiness during storage and germination. It is reported that type-*h* thioredoxins accumulate in the nucleus of developing wheat seed tissues subjected to oxidative stress [20]. This study identifies and characterizes the target proteins of TRX *h1* in European beech seed axes and explores their potential roles in redox regulation and seed longevity. By investigating proteins that interact with TRX *h1*, we sought to uncover how redox networks contribute to seed viability, metabolism, and oxidative stress protection during long-term storage. In addition to identifying these targets, we experimentally assessed the total thiol content of the embryonic axes in freshly harvested and long-term stored seeds, providing functional evidence that the reduced thiol pool on which TRX *h1* acts changes during storage.

This research provides insights that are critical for developing improved storage protocols, supporting seed resilience against environmental stresses, and offering practical applications for forest conservation and restoration efforts. By advancing our understanding of the molecular mechanisms governing seed longevity, this study has significant implications for the conservation of species such as European beech, which face heightened environmental pressures due to climate change.

## 2. Materials and methods

### 2.1 Plant material collection

European beech seeds, which are categorized as intermediate storage seeds, were collected in 2020 from individual trees growing in Kórnik Arboretum (western Poland, 52°14′35″N, 17°05′33″E). These seeds were then dehydrated to 10%. Each lot was packed into a plastic container and stored at 4 °C. Fully developed embryos of mature seeds were isolated from intact seeds and imbibed in water overnight prior to TRX *h1* target protein identification. For the determination of thiol (SH) content, embryonic axes were obtained from two seed lots harvested from the same tree in Kórnik Arboretum: a freshly harvested lot and a lot stored for 15 years at −10 °C in closed plastic boxes at 8–9% water content. The Kórnik Arboretum is owned and managed by the Institute of Dendrology, Polish Academy of Sciences. As the plant material was collected on the premises of the host institution, no specific permits were required to access the field site or to collect the seed material used in this study.

### 2.2 Mutagenesis and heterologous production of His-tagged TRX *h1*

Protein expression and purification were conducted in accordance with established protocols described in [21]. Briefly, the coding sequence of TRX *h1* (from *Pisum sativum*) was subjected to PCR mutagenesis via specific primers that facilitated the replacement of a cysteine residue with serine at the active site (see Table 1 for primer details). Then, the amplicon was cloned and inserted into the expression vector pETM-11 in which a His-tag was added to the N-terminal end of the protein. The *Escherichia coli* BL21 strain was then transformed with the resulting expression plasmid. The transformed bacteria were then cultivated at 37 °C until an O.D. 600 nm of 0.4 was obtained, at which point protein expression was induced with 0.4 mM isopropyl β-D-1-thiogalactopyranoside (IPTG) for 4 hours. The recombinant protein His-CXXS was purified by Co^2+^ affinity chromatography in accordance with the manufacturer's instructions (HiTrap Talon, Cytiva). *P. sativum* was used as a bait species because, at the time of the study, the European beech genome was in an early draft version, and PCR primers designed based on its sequences were unreliable, limiting its use for protein interaction analyses. This approach allowed us to exploit a well-characterized TRX *h*1 sequence from a related plant species to identify potential targets in European beech seeds.

**Table 1:** Oligonucleotides used for directed mutagenesis and heterologous expression of TRX *h1* in *Escherichia coli*. Restriction sites are underlined (PeahN, NotI; PeahC, BamHI).

Name	Sequence	Use
PeahN	5’‑AGACCATGGCAGGTTCATCAGAAGAGG-3’	Cloning into pETM-11
PeahC	5’‑TTGGATCCCTAAGCATTAGATGAAGCCAC-3’	
PsTRXh1-CS-F	5’-TTGGTGTGGACCATCCCGTTTCATTGCACC-3’	Mutagenesis
PsTRXh1-CS-R	5’-GGTGCAATGAAACGGGATGGTCCACACCAA-3’	

### 2.3 Protein study with LC-MS/MS analysis

#### 2.3.1 Protein isolation.

The embryonic axes of European beech were homogenized in a cooled mortar with liquid nitrogen and using 200 mg per sample (three biological replicates). The resulting homogenate was subsequently suspended in extraction buffer containing 50 mM Tris-HCl (pH 7.8), 10 mM KCl, 20% glycerol, and a cOmplete (TM) protease inhibitor cocktail (Roche, Basel, Switzerland) to extract the soluble protein. The protein content was measured using the Bradford method [22].

#### 2.3.2 Target capture and purification.

To capture the TRX *h1* target, protein extracts from embryonic axes were incubated with His-CXXS for 30 minutes at room temperature (RT) under gentle agitation. Following incubation, the CXXS-target complexes were purified using a pull-down process with Dynabeads (Invitrogen, Waltham, MA, USA) that binds His-tagged proteins and according to the manufacturer's instructions. Following the isolation of the target proteins, a Western-blotting analysis was performed using an anti-TRX h1 antibody (rabbit) at a 1:5000 dilution to detect His-CXXS complexed with the target proteins. The purified complexes were then incubated with 50 mM DTT for 30 minutes at RT to reduce the disulfide bridge and separate targets from His-CXXS. DTT was removed by spin dialysis using Zeba™ Spin Desalting Columns (7K MWCO, Thermo Fisher Scientific) equilibrated with Dynabeads binding buffer. To isolate the TRX *h1* targets, Dynabeads were used again for the pull-down process, but this time, the wash fraction containing the target was collected instead of the elution fraction, which contained only His-CXXS. Targets were then incubated overnight with 15% trichloroacetic acid at −20°C and centrifuged at 4°C for 20 minutes at 15000 × g. The pellet was washed twice with 80% (v/v) ice-cold acetone before liquid chromatography‒tandem mass spectrometry (LC-MS/MS) analysis.

#### 2.3.3 LC‒MS/MS analysis of proteins.

The precipitated target proteins were analyzed using the methods previously described by Torres-Romero et al. [23]. To reach a final concentration of 5 mM, dithiothreitol was added to the samples followed by a 30 minute incubation at 60 °C. Next, iodoacetamide was added to a final concentration of 10 mM, and samples were incubated for 30 minutes at room temperature in the dark. Enzymatic digestion was performed by incubating the solution with trypsin at a 1:40 ratio (trypsin) overnight at 37°C. After digestion, 1 µg of protein was analyzed using a Tandem Quadrupole Time-of-Flight mass spectrometer (AB/Sciex TripleTOF5600 Plus) connected to a Nanospray III Ion Source (AB/Sciex) and a nano-HPLC system (Eksigent Ultra 2D). Peptide separation involved removing impurities on an isocratic precolumn (C18 PepMap100 column NAN75-15-03-C18-PM, Thermo Fisher Scientific) with a solvent consisting of 0.1% formic acid and 5% (v/v) acetonitrile, which was delivered at a flow rate of 3 µl/min for 10 minutes. The peptides were then transferred to the analytical column through an integrated electrospray emitter (New Objective PicoFrit column, 75 µm internal diameter × 250 mm, packed with Reprosil-PUR 3 µm) and separated using a linear gradient of solvent B from 5% to 35% over 60 minutes at a flow rate of 250 nl/min. Solvent A was composed of 0.1% (v/v) formic acid, and solvent B was acetonitrile with 0.1% (v/v) formic acid. The ion source parameters were set as follows: ISVF = 2600, GS1 = 20, and CUR = 25. Data acquisition was conducted using the Data-Dependent Acquisition (DDA) method starting with a high-resolution TOF-MS scan over a mass range of 400–1250 m/z, followed by MS/MS scans of 50 ion candidates per cycle, which covers a mass range of 230–1500 m/z with dynamic background subtraction when operating in high-sensitivity mode. The ion accumulation times were set to 250 ms for MS and 65 ms for MS/MS. Protein identification was performed using ProteinPilot v5.0.1 software (Sciex) with the Paragon algorithm and a database of *Acer yangbiense* in FASTA format combined with the Sciex contaminant database from www.uniprot.org. This analysis was performed in the Proteomic Service of IBVF CSIC-US; Seville, Spain.

### 2.4 Western blot

To assess the redox state of the TRX *h1*-target interactions, the His-CXXS-bound protein samples from the embryonic-axis extracts were resolved under non-reducing and reducing conditions. For the non-reducing condition, samples were prepared in SDS-PAGE loading buffer without a reducing agent, leaving disulfide-linked TRX *h1*-target complexes intact; for the reducing condition, 50 mM DTT was added to cleave the mixed-disulfide bonds and release free TRX *h1*. Protein samples (20 µg) were separated on a 12% SDS-PAGE gel and transferred to a polyvinylidene fluoride membrane (Immobilon-P; Merck Millipore, Burlington, MA, USA) at 350 mA for 1 h using a Mini Trans-Blot Cell (Bio-Rad, Hercules, CA, USA). Membranes were blocked with 5% non-fat milk and incubated with a mouse anti-TRX *h1* antibody at a 1:5000 dilution, followed by an anti-mouse IgG secondary antibody conjugated with horseradish peroxidase at a 1:5000 dilution. Bands were visualized by enhanced chemiluminescence Immobilon Western Chemiluminescent HRP Substrate (Merck Millipore) and imaged on a G:BOX Chemi XRQ system (Syngene).

### 2.5 *Determination of total thiol content*

Total thiol (SH) groups were quantified in the embryonic axes of freshly harvested seeds and of seeds stored for 15 years to determine whether long-term storage was associated with changes in the thiol redox status of European beech seed axes. Samples were extracted in 0.02 M EDTA. SH groups were assayed with Ellman's reagent (DTNB). 0.001 M DTNB in methanol was added to 20 µL of sample in a final volume of 200 µL and absorbance was read at 412 nm (Infinite 200 PRO, Tecan). Under these conditions the reaction mixture contained approximately 79% (v/v) methanol, in which proteins are largely precipitated; the assay therefore reports the pool of thiols accessible to DTNB rather than the complete protein thiol pool, and the values are given as total thiols without subtraction of an acid-soluble fraction. For each sample, a paired blank without DTNB (DTNB replaced by an equal volume of buffer) was run to correct for the intrinsic absorbance of the extract; the DTNB-free value was subtracted from the corresponding reading. Thiol concentrations were calculated from the slope of a reduced glutathione (GSH) standard curve (0–200 µM, prepared with DTNB; R² = 0.988), taking the reagent blank of the sample plate as the zero point. Total thiol content is expressed per embryonic axis as GSH equivalents, obtained from the in-well concentration by correcting for the ten-fold dilution of the extract in the assay, the extraction volume and the number of axes per replicate. Three biological replicates per lot, each comprising ten embryonic axes, were analyzed; means were compared by Welch's t-test.

### 2.6 Cross-species comparison of TRX *h1* targets

The European beech TRX *h1* targets were compared with those previously identified in the embryonic axes of Norway maple (*Acer platanoides L.*) and sycamore maple (*Acer pseudoplatanus L.*) using the same His-CXXS trapping approach [21]. Because the three species do not share a common annotated genome, proteins were matched across species at the level of protein annotation, after normalization of names (lowercasing and removal of redundant whitespace and trailing punctuation). Of the 161 beech targets, 158 carried unique annotations and were used for the comparison; the remaining three corresponded to additional accessions sharing an annotation already present in the list. Shared and species-specific targets were determined and visualized as a Venn diagram in R 4.5.3 [24] using the ggVennDiagram package, version 1.5.7[25].

## 3 Results and discussion

Our study reveals a set of proteins that interact with TRX *h1* in European beech seeds and sheds light on the redox regulatory mechanisms that influence seed longevity and storage behavior. TRX *h1* is central to redox homeostasis and modulates protein activity via reversible disulfide bond formation, which is a process that is critical during seed maturation, dehydration, and storage. Given that redox imbalances are tightly linked to oxidative damage, especially in seeds subjected to long-term storage, our findings suggest a potential involvement of TRX *h1* in maintaining seed viability. Below, we discuss the implications of TRX *h1* targets for cellular processes and their potential influence on seed storage and longevity. Members of the type-*h* TRX subfamily are located in the cytoplasm, mitochondria, endoplasmic reticulum, and plasma membrane [26]. In this study, we highlight the potential involvement of TRX *h1* in determining seed storage capacity. Redox regulation, in addition to differences between seed types, plays a crucial role in determining optimal seed storage conditions and ensures the preservation of genetic resources in gene banks.

To relate the redox-regulatory network defined by the TRX *h1* targets to the physiological status of the seed thiol pool, we quantified total thiol content in the embryonic axes of fresh and long-term stored beech seeds. Total thiols were approximately 2.2-fold lower in aged than in fresh axes (21.0 ± 0.5 vs 45.9 ± 1.3 nmol GSH equivalents axis^-1^; Welch's t-test, p = 0.0002, *n* = 3; Fig 1). These data indicate that storage was accompanied by a marked decline in the reduced-thiol pool of the embryonic axes, the pool on which thiol-disulfide exchange reactions mediated by the TRX *h1* system operate.

To selectively identify proteins that form complexes with TRX *h1*, we used a modified version of the protein in which a cysteine residue was substituted with serine. This allowed us to isolate only those proteins that directly interact with TRX *h1*. Our analysis focused on seeds categorized as intermediate, which show reduced germination capacity over time but can withstand dehydration. Prior to mass-spectrometric identification, Western blotting supported the formation of redox-sensitive complexes between TRX h1 and embryonic-axis proteins: under non-reducing conditions, the anti-TRX h1 antibody (1:5000) detected a ladder of higher-molecular-mass mixed-disulfide complexes between the His-CXXS trapping mutant and its targets, which collapsed to free TRX h1 upon DTT treatment (Fig 2). This DTT-reversible pattern indicates that the captured proteins are bona fide redox partners of TRX h1 rather than co-purifying contaminants, thereby confirming that target capture proceeded through redox-active, DTT-reducible disulfide bonds; the identity of the individual partners was established by the subsequent DDA analysis. Using data-dependent acquisition (DDA) methods, we generated a library of proteins that form complexes with TRX *h1* in European beech seed axes and we identified 161 proteins as detailed in S1 Table. The identified proteins belong to several functional categories, including cellular processes (8), genetic information processing (66), metabolism (47), environmental information processing (7), unclassified proteins (33), as shown in Fig 3.

**Fig 1:**
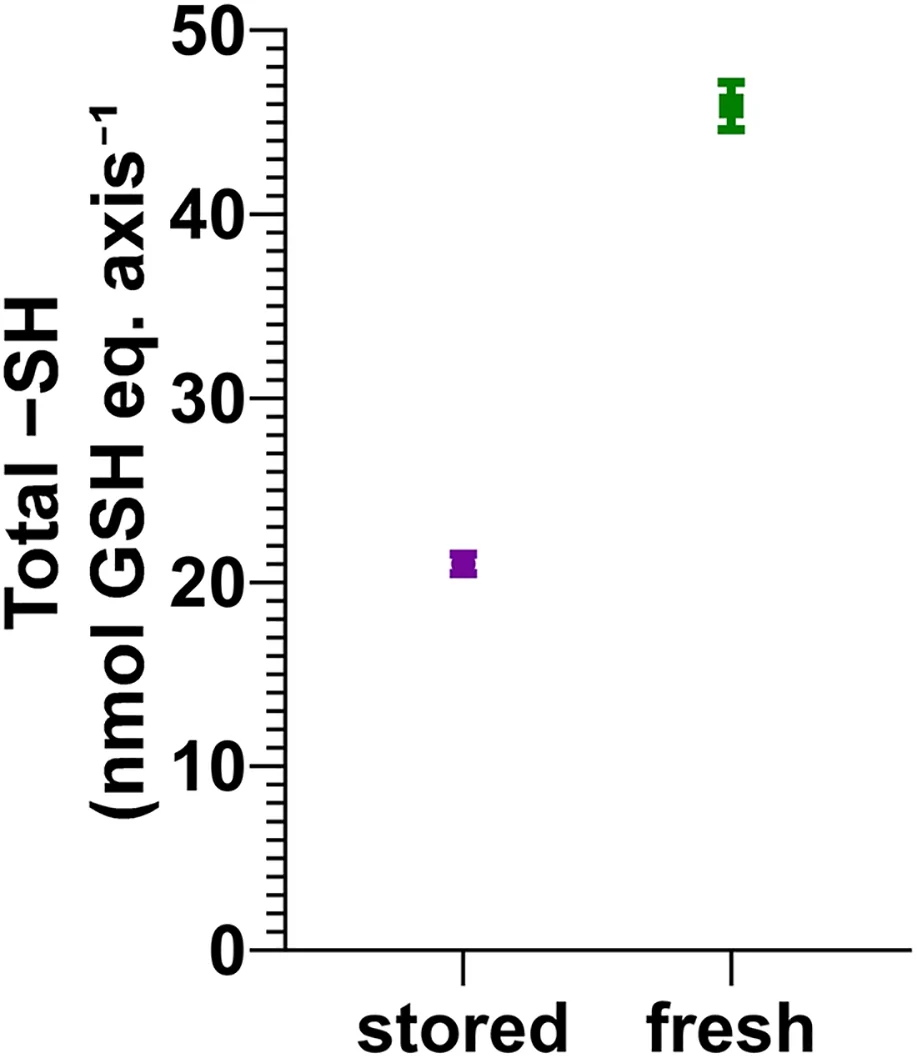
Total thiol content of embryonic axes from freshly harvested and long-term stored European beech seeds. Thiol (SH) groups were quantified with Ellman's reagent (DTNB), calculated against a reduced glutathione (GSH) standard curve and expressed per embryonic axis as GSH equivalents. Fresh, freshly harvested seeds; Stored, seeds stored for 15 years. Values are means ± SD (n = 3 biological replicates of ten axes each); total thiols were approximately 2.2-fold lower in stored than in fresh axes (Welch's t-test, p = 0.0002).

**Fig 2:**
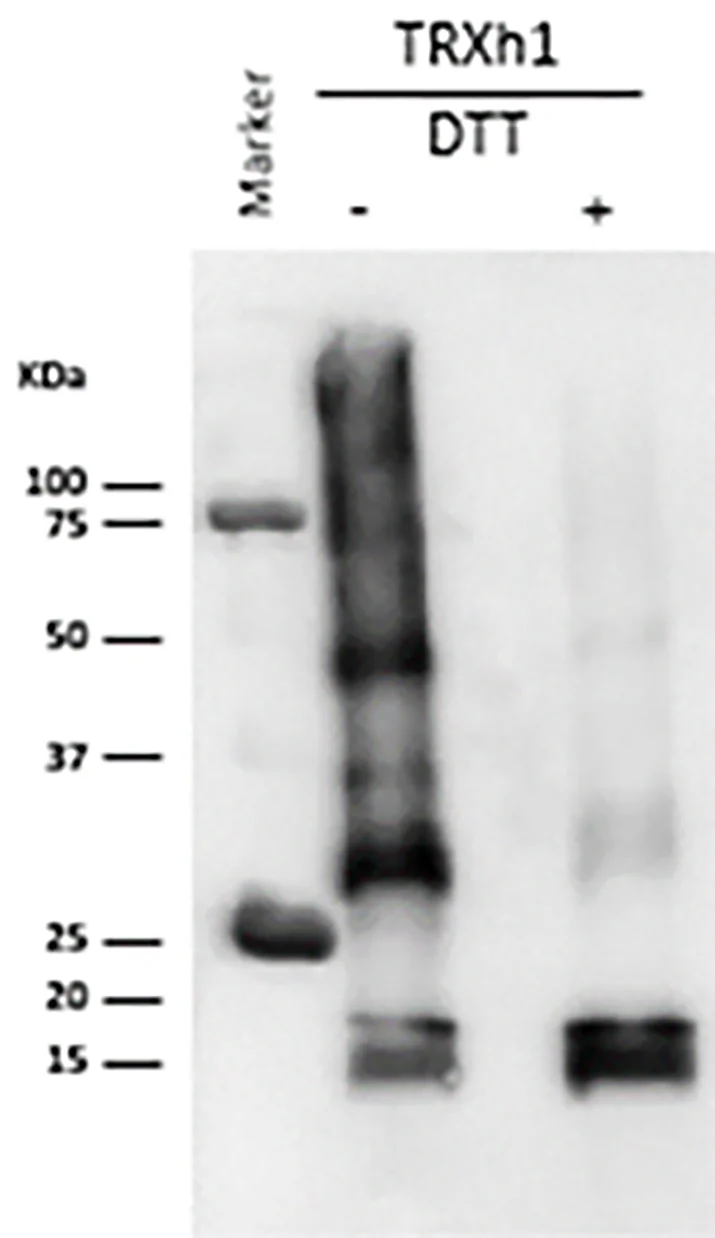
Validation of TRX h1–target trapping by Western blot. Embryonic-axis protein extracts of European beech were incubated with the His-tagged trapping mutant His-CXXS (Cys → Ser) of TRX *h1* and resolved under non-reducing (− DTT) and reducing (+ DTT) conditions; the membrane was probed with an anti-TRX *h1* antibody (1:5000). Under non-reducing conditions, TRX *h1* is detected as a ladder of higher-molecular-mass bands corresponding to mixed-disulfide complexes with target proteins, whereas DTT treatment reduces these disulfide bonds and releases free TRX *h1* (~ 13–15 kDa), collapsing the high-mass signal. This confirms that the captured proteins interact with TRX *h1* through redox-active, DTT-reducible disulfide bonds. Molecular masses (kDa) are indicated on the left.

**Fig 3:**
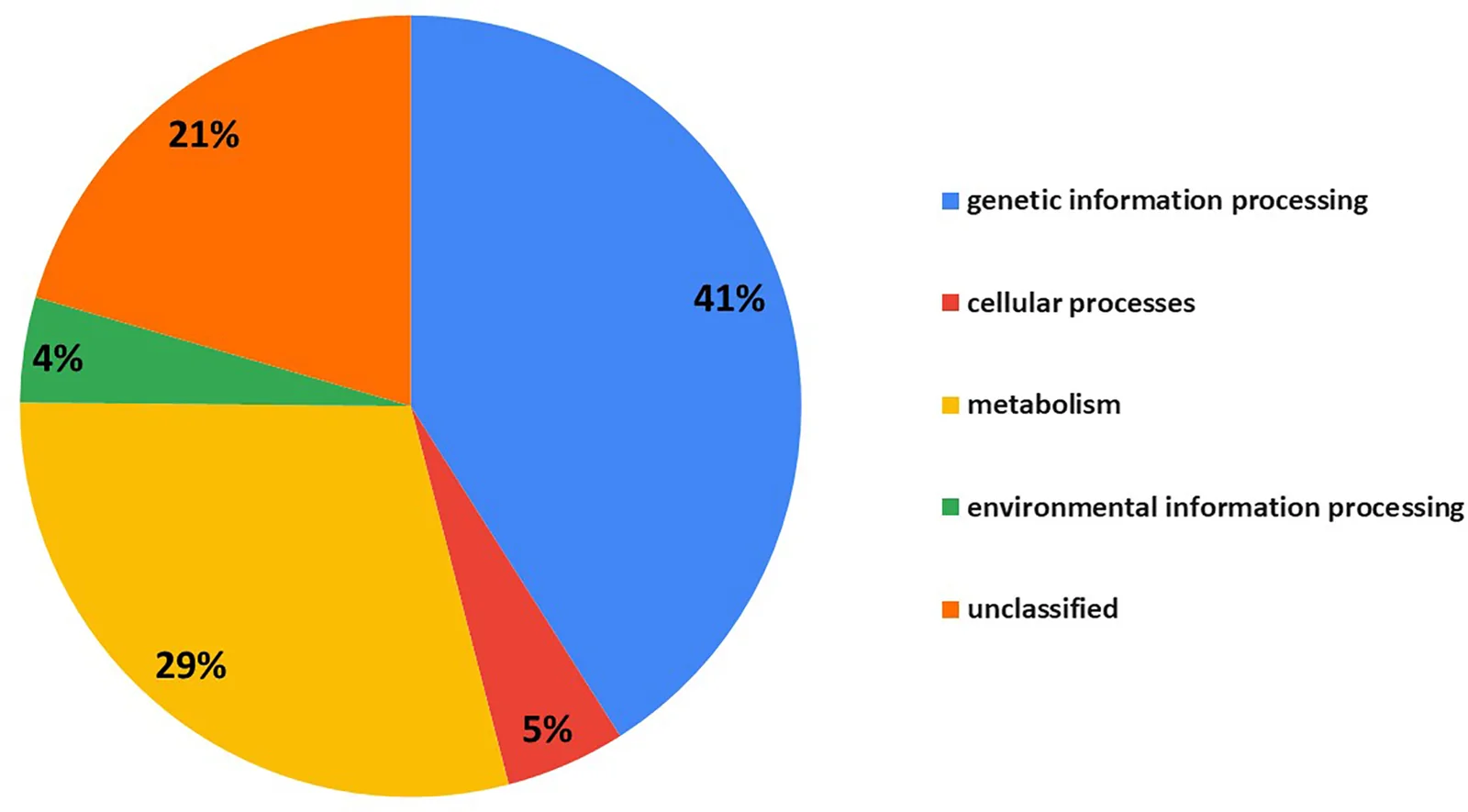
Protein functions of TRX *h1* target proteins in mature European beech seed axes.

### 3.1 Genetic information processing

We identified 66 proteins involved in genetic information processing with a particular focus on proteins that are potential targets of TRX *h1*. The dominant proteins were chaperones and heat shock proteins (HSPs) as well as proteins involved in protein synthesis, folding, transport, and degradation. Additionally, we identified proteins related to RNA metabolism and the cell cycle (S1 Table). These findings highlight the importance of TRX-mediated redox regulation in maintaining protein homeostasis and ensuring proper cellular functions in the embryonic axes of beech seeds.

### 3.2 Heat shock proteins and chaperones as thioredoxin targets

In our study, the beech seed axes targeted by TRX *h1* included HSPs from the HSP70 and HSP90 families. These molecular chaperones are critical for ensuring proper protein folding, assembly, translocation, and degradation, particularly under stress conditions. Chaperones were also identified as potential targets for TRXs in studies on spinach and poplar [27] and in *Escherichia coli* [28]. The identification of these HSPs as thioredoxin targets suggests that redox regulation may play a crucial role in modulating their activity during both stress responses and normal cellular processes [29]. Initially recognized in response to heat shock [30], HSPs are now known to be upregulated by various environmental and physiological stresses such as cold, UV light, mechanical injury, tissue remodeling, and pathogen invasion. TRX-mediated redox modifications can modulate the function of these HSPs and increase their ability to manage cellular stress and maintain homeostasis [29]. The regulation of HSP90 by TRX has already been extensively described in humans and animals [e.g., [31–33]]. However, in plants, many questions remain regarding the importance of HSP90 regulation by TRX. This topic offers a broad scope for further research.

### 3.3 DnaJ homolog and ribosome-associated complex

Another significant TRX *h1* target we identified is the DnaJ homolog subfamily C member 2-like protein. This protein acts both as a cytosolic chaperone and a nuclear chromatin regulator. As part of the ribosome-associated complex (RAC), it assists in the folding of nascent polypeptides, whereas in the nucleus, it regulates gene expression by facilitating the activation of polycomb-repressed genes. The identification of this protein as a TRX *h1* target suggests that redox regulation may influence its dual roles in protein folding and gene regulation, particularly through TRX-mediated modifications that impact its interactions with other proteins involved in these processes. Another DnaJ protein from the C subfamily, member 9, was also identified as a target protein for TRXs in studies on human TRXs [34].

Similarly, NudC proteins and J-domain proteins (JDPs), which have also been identified as potential TRX *h1* targets, are critical for protein quality control. The modulation of JDPs by TRX could impact their interactions with HSP70s and affect the broader protein-folding machinery within the cell [35].

### 3.4 Nascent-Polypeptide-Associated Complex (NAC)

The NAC, another potential TRX *h1* target, is a heterodimeric complex associated with ribosomes. NAC is believed to protect nascent polypeptide chains as they exit the ribosome. TRX-mediated redox regulation of NAC may affect its role in protein translocation either by inhibiting or facilitating the transport of newly synthesized proteins to specific cellular compartments [36]. These findings suggest that TRX regulation of NAC could be crucial for maintaining protein homeostasis, particularly during stressful conditions that require rapid adjustment of protein synthesis and transport.

### 3.5 Protein Disulfide Isomerases (PDIs) and their redox regulation

In plants, protein disulfide isomerases (PDIs) are key enzymes that assist in the folding of nascent and misfolded proteins by catalyzing disulfide bond formation. Our identification of PDIs as TRX targets could be particularly important during heat stress and other conditions that induce the unfolded protein response (UPR); PDIs, including members of the PDI-M family in *Arabidopsis*, are upregulated in response to such stress [37]. The fact that PDIs have been found among TRX *h1* targets raises the question of a putative redox regulation of these proteins in plants.

### 3.6 Ribosomal proteins, elongation factors, and initiation factors as thioredoxin targets

Among the identified potential TRX *h1* target proteins were several ribosomal proteins, elongation factors, and initiation factors, which are key components of the translation process. These include 60S ribosomal L2, L12, P0, P1, P3-like proteins, elongation factor 1-beta, and eukaryotic translation initiation factors (eIF3D and eIF4C).

The identification of numerous translation-related proteins, chaperones, and stress-response proteins targeted by TRX *h1* highlights the central role of redox regulation in controlling key cellular processes in beech embryonic axes. TRX *h1*-mediated modifications may allow the protein synthesis machinery of the embryo to be fine-tuned during critical stages of development and ensure that protein homeostasis is maintained even under fluctuating environmental conditions. Further research into the specific redox modifications of these proteins will be essential for understanding the full scope of the regulatory role of TRX *h1* during seed development, storage and germination.

The methyl CpG-binding protein (MBD) binds to methylated DNA via its MBDs, which is a conserved sequence motif of 70 amino acids. To date, 13 putative MBD-containing genes (AtMBD1–13) have been identified in the model plant *Arabidopsis thaliana* [38]. The identification of the MBD protein, which is involved in chromatin remodeling and DNA methylation, suggests that TRX *h1* may influence epigenetic regulation during seed maturation and storage. Epigenetic changes, including DNA methylation, have been shown to play a role in seed dormancy and stress responses and further emphasizes the importance of TRX *h1* in modulating gene expression in response to environmental cues.

### 3.7 Cellular and environmental process signaling

In this category, a total of 15 proteins were identified that are primarily associated with various cellular processes. The main categories included proteins involved in the following: calcium signaling and transport, such as calreticulin (CRT) and calmodulin, metabolic and enzymatic regulation, cytoskeleton organization and cell movement, and proteins related to stress response and defense mechanisms. Additionally, some of the identified proteins play roles in embryonic development and protein synthesis regulation (S1 Table). Calcium serves as a key secondary messenger in cell signal transduction pathways in both plants and animals. Furthermore, it regulates various cellular functions by binding to specific calcium-binding proteins. One such protein that we identified in our study is calmodulin, which plays a crucial role in regulating plant growth and development and in resistance mechanisms to various biotic and abiotic stresses [39]. Calmodulins regulate the expression of HSP genes under stress conditions [40,41]. The identification of a calmodulin family protein is important because we have identified numerous HSPs, as described above.

Calreticulin (CRT) is found in the endoplasmic reticulum of both plant and animal cells where it functions as a ubiquitous molecular chaperone. The antioxidant role of plant calreticulin in alleviating oxidative stress and enhancing stress tolerance has been demonstrated in transgenic plants. The overexpression of calreticulin from *Triticum aestivum* has led to increased drought resistance in tobacco plants cultivated under limited water conditions [42,43]. Research has shown that the C-domain of TaCRT1 is crucial for its ability to tolerate various stresses in plants [42]. The findings from several antioxidant assays indicate that, in contrast with wild-type plants, transgenic plants with CRT overexpression have significantly elevated levels of antioxidant enzymes including superoxide dismutase (SOD) [42,43]. Interestingly, in the context of our results, we observed the presence of Cu/Zn SOD in the proteome of the target proteins of TRX *h1*. Studies indicate that in animal cells, the upregulation of CRT is correlated with an increase in the levels of TRXs and their related antioxidant enzymes at the protein and mRNA levels [43–45]. In plants, this relationship between CRTs and TRXs has not been well described, although CRTs have previously been identified as target proteins for TRXs in *Medicago truncatula* seeds [46].

Translationally Controlled Tumor Proteins (TCTPs) in plants play diverse roles in stress tolerance [47], act as chaperone-like proteins in thermotolerance [48], aid in calcium binding [49], and contribute to oxidative stress defense [50]. Their ability to interact with proteins and protect against denaturation, as well as their involvement in calcium-mediated signaling pathways, highlights their importance in responding to abiotic stresses such as drought and heat [51]. TCTPs also show potential in biotic stress responses including an improved tolerance to pests and viruses [52]. Nevertheless, the role of TRX *h1* in regulating TCTP activity in plants remains unknown.

### 3.8 Energy metabolism

In our study, we identified 47 TRX target proteins related to metabolism in European beech seed axes, which play essential roles in energy metabolism, particularly through the regulation of carbohydrate, amino acid, and lipid metabolic pathways. TRXs modulate the activity of key enzymes through redox-dependent changes and influence pathways critical for energy production, growth, and stress responses [27,53,54]. The identified target proteins highlight the extensive influence of TRX across various metabolic processes. In carbohydrate metabolism, several TRX-regulated enzymes, such as fructose-bisphosphate aldolase (FBA), malate dehydrogenase (MDH), and phosphoglycerate kinase (PGK), are essential for glycolysis and the TCA cycle, which are vital for energy production [16,55]. For example, FBA has been identified as a redox-sensitive protein in sycamore seeds, where oxidative modifications can influence its glycolytic activity, especially under dehydration stress. In sycamore seeds, the lack of “metabolic shutdown” due to oxidative stress could lead to continuous metabolic activity even under low-water conditions, which would increase the risk of energy depletion and reduce seed viability [55]. In contrast, Norway maple seeds exhibit tighter metabolic control, which likely contributes to their desiccation tolerance and capacity for long-term storage. During dormancy in Norway maple seeds, abscisic acid (ABA) downregulates FBA and potentially limits energy availability and delays germination [56]. MDH, another TRX *h1* target, catalyzes the oxidation of malate in the TCA cycle and serves as a critical component in cellular respiration. Several proteomic studies suggest that mitochondrial MDH is a TRX target in plants [27,53,54]. In addition, recent studies have identified MDH as a TRX *h1* target in the seed axes of sycamore (*Acer pseudoplatanus*), which further supports the role of TRXs in regulating energy metabolism during seed development and storage [21]. In sycamore seeds, MDH levels are modulated by endogenous gibberellins and ABA during dormancy release, which suggests a complex regulatory interplay between redox status and hormone signaling [57]. Similar findings occurred in *Coffea arabica* and *Coffea eugenioides*, where MDH expression decreases in desiccation-tolerant seeds and this supports our results on MDH regulation in beech seeds [58]. In amino acid metabolism, TRX regulates enzymes such as 5-methyltetrahydropteroyltriglutamate-homocysteine S-methyltransferase, glycine cleavage system H protein, and adenosylhomocysteinase, which are involved in amino acid biosynthesis and degradation. This regulation ensures a balanced supply of amino acids, which are essential for protein synthesis, cellular growth, and the response to stress. Amino acid availability is crucial during seed development and germination, where metabolic flexibility can support resilience under fluctuating environmental conditions [59]. In lipid metabolism, TRX influences enzymes, such as acyl carrier proteins and AAA superfamily proteins, both of which are involved in lipid biosynthesis and membrane integrity. Through these enzymes, TRX helps maintain lipid homeostasis, which is essential for both energy storage and structural stability, particularly as seeds undergo dehydration and later rehydration during germination. Proper lipid regulation is crucial to ensure that seed membranes remain functional and allows seeds to recover from the desiccation required for long-term storage [60].

### 3.9 Redox regulation and oxidative stress

Our study revealed that key proteins, such as glutathione peroxidase (GPX) and glutaredoxin (GRX), interact with TRX *h1* and contribute to redox homeostasis in European beech seed axes. These proteins play a central role in mitigating oxidative stress, which is a critical factor in seed aging and viability loss during storage [61]. GPX reduces harmful peroxides, whereas GRX maintains a protein thiol balance that protects cellular proteins from oxidative damage, which can lead to protein aggregation and functional loss [62]. The interactions of TRX *h1* with these proteins suggest that enhancing the redox balance could improve seed longevity [21].

SODs, particularly those from the Cu/Zn-SOD family, serve as a first line of defense against ROS by converting superoxide radicals into hydrogen peroxide [63]. In beech seeds, SOD activity has been linked to ROS detoxification during storage with increased enzyme levels noted under stress conditions. However, excessive ROS levels can overwhelm these defenses and lead to a loss of viability, particularly under prolonged or extreme storage conditions [61].

The results of a study by [64] further emphasize the importance of superoxide metabolism during germination and growth and suggests that SOD activity and subsequent hydrogen peroxide production are essential for early cell wall modification and the initiation of seed germination.

The approximately 2.2-fold decrease in total thiol content per axis indicates that the axes shift towards a more oxidized state during storage. This change reflects oxidation of reactive cysteine residues, which may arise from reversible disulfide formation, S-glutathionylation and irreversible higher oxidation, potentially including cysteine residues within some of the redox-sensitive candidate TRX *h1* targets identified here (e.g., GAPDH, FBA, MDH, GPX, GRX, Cu/Zn-SOD). The reversibly oxidized fraction is the natural substrate of the TRX *h1* system, whereas irreversibly oxidized thiols lie beyond its reach. This is consistent with the recognized role of the protein thiol redox state as a marker of storage and desiccation tolerance in seeds [62] and points to a possible contribution of TRX *h1*-dependent redox maintenance to beech seed longevity. However, these measurements reflect the overall oxidative/reductive environment of the embryonic axes during storage and do not by themselves demonstrate a specific role for a particular thioredoxin; further functional studies are required to confirm the involvement of TRX h1 in maintaining seed viability.

This interpretation is further supported by our previous study on European beech seeds, in which protein-bound thiols were shown to be highly dynamic during development and storage. In that work, non-protein thiols remained comparatively stable during storage, whereas protein thiols declined, indicating that the protein thiol pool represents a particularly storage-sensitive component of the redox system in beech seeds [16]. The present results extend this earlier observation by linking storage-related changes in the total reduced thiol pool with the identification of candidate TRX *h1*-interacting proteins involved in metabolism, antioxidant defense and protein homeostasis.

In our previous studies, we identified TRX *h1* target proteins in the embryonic axes of Norway maple and sycamore maple [21]. Therefore, we compared our new findings from European beech with TRX *h1* targets across seeds with different desiccation tolerance strategies as follows: Norway maple (orthodox), sycamore maple (recalcitrant), and European beech (intermediate). Notably, the following six TRX *h1* target proteins were conserved across all three species: phosphoglucomutase (alpha-D-glucose-1,6-bisphosphate-dependent), Cu/Zn SOD, nucleoside diphosphate kinase, GPX, 60S acidic ribosomal protein P0, and a nonspecific lipid-transfer protein. Additionally, European beech shares 32 TRX *h1* targets with only sycamore maple and three with only Norway maple (Fig 4), which reflects potential differences in protein redox regulation linked to desiccation tolerance strategies. The greater number of shared targets with the recalcitrant sycamore maple may suggest that intermediate seeds, such as European beech, retain redox mechanisms that are more aligned with desiccation-sensitive species. This finding provides valuable insights into the evolution of redox regulation across different seed categories and their potential adaptation to varying environmental conditions.

**Fig 4:**
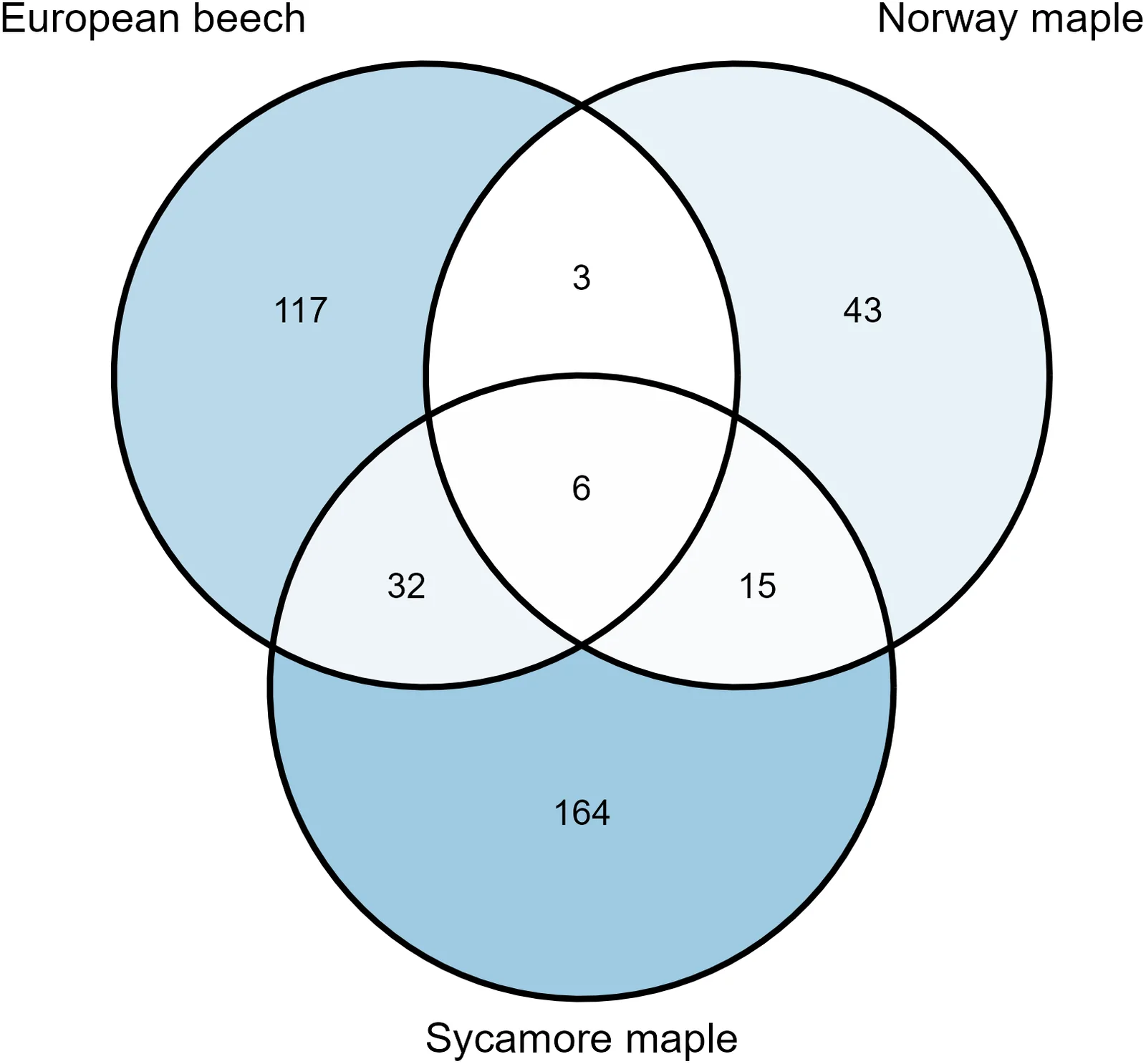
Venn diagram illustrating the number of shared and unique proteins identified as TRX *h1* targets in the embryonic axes of seeds from three tree species: European beech, Norway maple, and sycamore maple. The diagram presents a synthesis of data from this study combined with previously published findings by Fuchs et al. [21].

## Conclusions

Our findings highlight the complexity of regulatory pathways influenced by TRX h1 in European beech seeds and suggest its potential involvement in maintaining seed viability and resilience during storage. The identified pathways collectively support essential physiological processes that protect seeds from environmental stresses and promote longevity. Stress response and protein quality control: TRX h1 interacts with HSPs and other chaperones, which are essential for protein stability, especially under dehydration and oxidative stress. This pathway emphasizes the function of TRX h1 in managing protein folding and stability, which safeguards seeds from environmental stressors. Calcium signaling modulation: TRX h1 regulation of calcium-binding proteins, including calmodulin and calreticulin, suggests that calcium signaling plays a central role in coordinating responses to various stresses. This pathway may also intersect with protein quality control mechanisms, where calmodulin influences HSP expression and further enhances seed resilience. Epigenetic and gene expression regulation: The targeting of MBD proteins by TRX h1 implies that epigenetic modifications, such as DNA methylation, are involved in the stress response and developmental transitions. This pathway highlights the potential role of TRX h1 in modulating gene expression, which is crucial for maintaining dormancy and controlling germination timing. Energy metabolism and the TCA cycle: TRX h1 interaction with key metabolic enzymes supports its role in energy regulation within the seed. By modulating glycolysis and TCA cycle enzymes, such as MDH and FBA, TRX h1 may enhance efficient energy utilization during storage and dormancy and prepare seeds for successful germination. Oxidative stress defense: TRX h1 may contribute to antioxidant defense by regulating ROS-detoxifying enzymes such as SOD, GPX, and GRX. This pathway is vital for countering oxidative damage, which is a primary cause of seed aging, and thereby preserves seed viability through balanced redox homeostasis. Protein synthesis and folding: The involvement of ribosomal proteins and elongation factors suggests that TRX h1 supports translational regulation. This pathway ensures efficient synthesis of protective proteins, especially under fluctuating storage and environmental conditions, and promotes cellular homeostasis. These interpretations are consistent with our biochemical observations: the captured proteins formed DTT-reducible disulfide complexes with TRX h1, confirming a redox-active interaction, and the total thiol content of the axes was markedly lower in long-term-stored seeds (2.2-fold), consistent with a more oxidized cellular environment during storage. The conservation of six TRX h1 targets across species with contrasting desiccation-tolerance strategies further points to a core redox module associated with seed longevity. Together, these pathways illustrate the potential multifaceted role of TRX h1 in seed physiology and highlight its importance for developing strategies to increase seed longevity and improve conservation practices (summarized in Fig 5). Further functional studies, including the analysis of individual TRX h1 targets and the manipulation of TRX h1 activity in seeds, are needed to confirm the specific contribution of this protein to beech seed viability and longevity.

**Fig 5:**
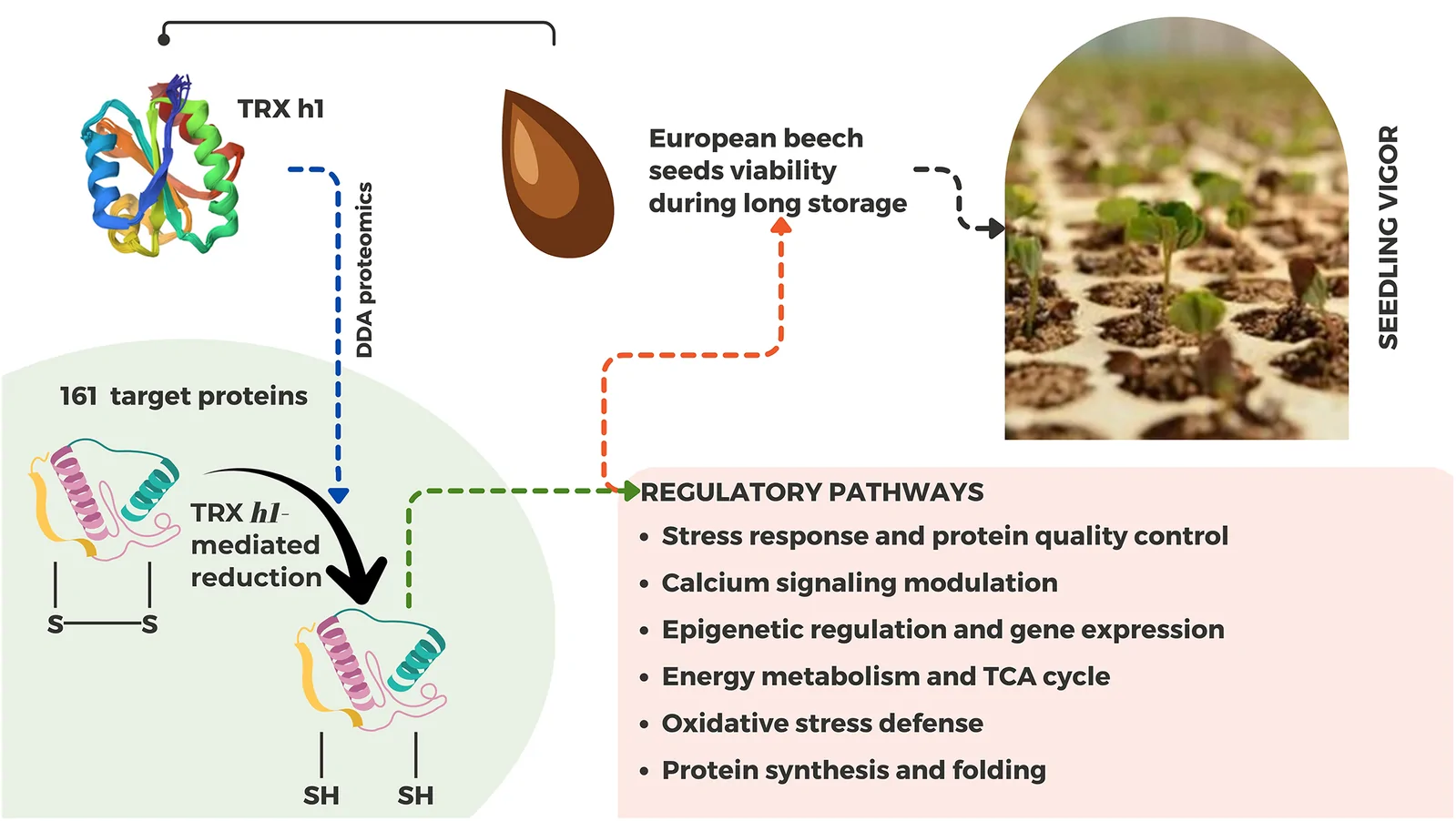
Schematic representation of the identified proteins involved in regulatory pathways influenced by TRX h1 in intermediate seeds. TRX h1 modulates multiple cellular processes, including oxidative stress defense, protein stability, calcium signaling, epigenetic regulation, and energy metabolism, all of which may contribute to seed viability and longevity.

## Supporting information

S1 Table Putative TRX h1 target proteins identified in the embryonic axes of European beech seeds. List of the 161 proteins captured with the His-CXXS trapping mutant and identified by LC–MS/MS, with accession numbers, protein annotations, functional category assignment and identification parameters.
(XLSX)

S1 File Original blot. (TIF)
